# The *xyl*-*doc* gene cluster of *Ruminiclostridium cellulolyticum* encodes GH43- and GH62-α-l-arabinofuranosidases with complementary modes of action

**DOI:** 10.1186/s13068-019-1483-y

**Published:** 2019-06-10

**Authors:** Mohamed Mroueh, Marion Aruanno, Romain Borne, Pascale de Philip, Henri-Pierre Fierobe, Chantal Tardif, Sandrine Pagès

**Affiliations:** 1 Aix Marseille Université, CNRS, LCB UMR7283, IMM-CNRS, 31 Chemin Joseph Aiguier, 13402 Marseille Cedex 20, France; 2 Institute of Microbiology, Lausanne University Hospital, Lausanne, Switzerland

**Keywords:** Arabinoxylan, Biomass degradation, α-l-Arabinofuranosidase, Characterization of enzymes, Substrate specificity, Cellulosomes, *Ruminiclostridium cellulolyticum*

## Abstract

**Background:**

The α-l-arabinofuranosidases (α-l-ABFs) are exoenzymes involved in the hydrolysis of α-l-arabinosyl linkages in plant cell wall polysaccharides. They play a crucial role in the degradation of arabinoxylan and arabinan and they are used in many biotechnological applications. Analysis of the genome of *R. cellulolyticum* showed that putative cellulosomal α-l-ABFs are exclusively encoded by the *xyl*-*doc* gene cluster, a large 32-kb gene cluster. Indeed, among the 14 Xyl-Doc enzymes encoded by this gene cluster, 6 are predicted to be α-l-ABFs belonging to the CAZyme families GH43 and GH62.

**Results:**

The biochemical characterization of these six Xyl-Doc enzymes revealed that four of them are α-l-ABFs. GH43_16_-1229 (RcAbf43A) which belongs to the subfamily 16 of the GH43, encoded by the gene at locus Ccel_1229, has a low specific activity on natural substrates and can cleave off arabinose decorations located at arabinoxylan chain extremities. GH43_10_-1233 (RcAbf43A_d2,3_), the product of the gene at locus Ccel_1233, belonging to subfamily 10 of the GH43, can convert the double arabinose decorations present on arabinoxylan into single O2- or O3-linked decorations with high velocity (*k*_cat_ = 16.6 ± 0.6 s^−1^). This enzyme acts in synergy with GH62-1234 (RcAbf62A_m2,3_), the product of the gene at locus Ccel_1234, a GH62 α-l-ABF which hydrolyzes α-(1 → 3) or α-(1 → 2)-arabinosyl linkages present on polysaccharides and arabinoxylooligosaccharides monodecorated. Finally, a bifunctional enzyme, GH62-CE6-1240 (RcAbf62B_m2,3_Axe6), encoded by the gene at locus Ccel_1240, which contains a GH62-α-l-ABF module and a carbohydrate esterase (CE6) module, catalyzes deacylation of plant cell wall polymers and cleavage of arabinosyl mono-substitutions. These enzymes are also active on arabinan, a component of the type I rhamnogalacturonan, showing their involvement in pectin degradation.

**Conclusion:**

Arabinofuranosyl decorations on arabinoxylan and pectin strongly inhibit the action of xylan-degrading enzymes and pectinases. α-l-ABFs encoded by the *xyl*-*doc* gene cluster of *R. cellulolyticum* can remove all the decorations present in the backbone of arabinoxylan and arabinan, act synergistically, and, thus, play a crucial role in the degradation of plant cell wall polysaccharides.

**Electronic supplementary material:**

The online version of this article (10.1186/s13068-019-1483-y) contains supplementary material, which is available to authorized users.

## Background

Arabinose-containing polysaccharides are found mainly in hemicelluloses and pectins. Arabinoxylan, the most abundant hemicellulosic component, is found in a wide range of plant species, hard wood, and annual plants [1]. Arabinoxylan is constituted of a linear backbone of β-(1 → 4)-linked d-xylopyranosyl units (Xyl*p*) decorated by α-l-arabinofuranosyl (Ara*f*) substituents attached through *O*-2 and/or *O*-3. In arabinoxylan, *O*-acetyl substitutions of xylose residues are common, but 4-*O*-methyl-d-glucuronic acid is also found [2]. The ratio Ara*f*/Xyl*p* in plant cell wall varies considerably, from 0.6 to 0.07 [3]. Some of the arabinose residues are linked to ferulic acids by ester bonds and the formation of ferulate dimers creates arabinoxylan–arabinoxylan cross-links. Pectins, a highly complex and heterogeneous group of polysaccharides, are composed of two types of regions: homogalacturonan and type I rhamnogalacturonan. Arabinan and arabinogalactan I are found in type I rhamnogalacturonan side chains. Arabinan backbone consists of α-(1 → 5)-linked-Ara*f* units branched at *O*-3 or *O*-2 with Ara*f* units and AGI consists of a β-(1 → 4)-d-galactopyranosyl backbone with *O*-3 substitutions of α-linked-Araf residues [4].

α-l-Arabinofuranosidases (α-l-ABFs), also called arabinoxylan-arabinofuranohydrolases (AXHs), are exoenzymes involved in the cleavage of α-(1 → 2), α-(1 → 3) or α-(1 → 5)-linked arabinosyl decorations in arabinoxylan, arabinan, arabinogalactan, and arabinoxylooligosaccharides (AXOS) [5–9]. α-l-ABFs are found in glycoside hydrolase (GH) families 2, 3, 43, 51, 54, and 62. They are divided into three types depending on their mode of action on arabinoxylan. AXHs-m cleave off arabinose from mono-substituted (α-(1 → 2) or α-(1 → 3)-linked) xylose residues. AXHs-d act on double-substituted xylose residues and remove either α-(1 → 2)-Ara*f* linkages or α-(1 → 3)-Ara*f* linkages. Finally, AXHs-m,d display a dual activity on mono-substituted and double-substituted xylose residues located at chain extremities and/or internal [6, 10]. α-l-ABFs able to cleave α-(1 → 5)-Ara*f* linkages catalyze the cleavage of terminal α-l-arabinofuranosyl residues on decorated or linear arabinan. Arabinosyl substitutions in hemicelluloses and pectins may hinder the catalytic interaction of the enzymes with the substrate backbone, and negatively affect the hydrolysis. Moreover, arabinosyl substitutions participate in the cross-linkage of polysaccharides within the plant cell wall. α-l-ABFs are, thus, required for the complete degradation of hemicelluloses and pectins, and exhibit a cooperative effect with other lignocellulose-degrading enzymes [11].

*Ruminiclostridium cellulolyticum* H10 (ATCC 35319), a non-ruminal, mesophilic cellulolytic bacterium [12], secretes carbohydrate-active enzymes (CAZymes) involved in the degradation of plant cell wall polysaccharides (http://www.cazy.org). Some of them, known as cellulosomal enzymes, bear a dockerin module and can interact with a non-catalytic scaffolding protein, CipC, carrying eight complementary cohesin modules, to form cellulosomes. Many of the well-characterized cellulosomal enzymes are encoded by the *cip*-*cel* operon (loci Ccel_0728 to Ccel_0739) or belong to the GH9 family [13–15]. The 26-kb *cip*-*cel* operon encodes essential subunits for the formation of cellulosomes and cellulose degradation [16, 17]. Another large gene cluster of 32 kb, called *xyl*-*doc* (loci Ccel_1229 to Ccel_1242), was identified and encodes secreted dockerin-bearing proteins which all exhibit a carbohydrate-binding module (CBM) predicted to target hemicelluloses (mainly CBM6) and a catalytic module putatively involved in the degradation of hemicelluloses (glycoside hydrolases GH2, 10, 27, 30, 43, 59, 62, and 95 and carbohydrate esterases CE1 and CE6) [18]. The expression of *xyl*-*doc* genes is activated by XydR, a response regulator, in the presence of straw, arabinoxylan, xylose, or arabinose [18]. The *R. cellulolyticum* genome analysis showed that the 6 cellulosomal putative α-l-ABFs are encoded exclusively by the *xyl*-*doc* gene cluster [19]. Four genes (loci Ccel_1229, Ccel_1231, Ccel_1233, and Ccel_1235) encode GH43 enzymes. Two genes (loci Ccel_1234 and Ccel_1240) encode GH62-containing proteins. The gene at locus Ccel_1240 encodes a putative bifunctional enzyme with one GH62 module and one CE6 module putatively involved in the hydrolysis of acetyl groups presents in xylan backbone. All these enzymes were detected in the cellulosomes [18, 19] and could drive the removal of arabinose decorations in arabinoxylans, an indispensable stage for complete hydrolysis. All of these enzymes were characterized biochemically and a mode of action was proposed for each of them based on generated three-dimensional models. In summary, these enzymes can remove all the Ara*f* decorations and acetyl substitutions that arabinoxylan can carry and are also active on arabinan. They play a crucial role in the degradation of these compounds.

## Methods

### Bacterial strains and plasmids

Genomic DNA from *R. cellulolyticum* ATCC 35319 (NCBI Reference Sequence: NC_011898.1) served as a template for amplification by PCR of the genes at loci Ccel_1229, Ccel_1231, Ccel_1233, Ccel_1234, Ccel_1235, and Ccel_1240 encoding the mature forms of the putative α-l-arabinofuranosidases. The list of primers used in this study is provided in Additional file 1: Table S1. The amplicons were cloned in pET22b(+) (Novagen) at *Nde*I/*Xho*I sites or in pET28b(+) (Novagen) at *NcoI/XhoI*, to introduce six histidine codons at the 3′ extremity of the coding sequence, except, for the gene encoding, the putative α-l-arabinofuranosidases GH43_29_-1231 which is cloned in the pGEX-5X-2 vector (Sigma-Aldrich) at *BamH*I/*Xho*I, to add a GST-tag (glutathion S-transferase) at the N-terminal extremity of the recombinant protein. In this case, the DNA sequence encoding the hexa-histidine tail was introduced in the primer 1231pGexrev. Positive clones were verified by DNA sequencing (Genewiz). The BL21(DE3) *Escherichia coli* strain was used as production strain for all the recombinant proteins, except for GH43_29_-1231 for which the DH5α (New England Biolabs) strain was used.

### Protein production and purification

Recombinant proteins were purified from 700 mL cultures in lysogenic broth medium supplemented with glycerol (0.85%) and the appropriate antibiotic (at the final concentration of 200 µg/mL for ampicillin and 100 µg/mL for kanamycin). After growth at 37 °C until A_600nm_ = 1.5–2, the cultures were cooled, and induction of the gene expression was performed overnight at 18 °C with 150 μM of IPTG (isopropyl β-d-1-thiogalactopyranoside). After 16 h of induction, the cells were harvested by centrifugation (3000×*g*, 15 min, 4 °C), resuspended in 30 mM Tris–HCl, pH 8.0, 1 mM CaCl_2_, supplemented with few milligrams of DNase I (Roche Applied Science), and broken in a French press. The crude extract was centrifuged 15 min at 10,000*g* at 4 °C and loaded on 2–5 mL of nickel–nitrilotriacetic acid resin (Qiagen) equilibrated in the same buffer. The proteins of interest were eluted with 100 mM imidazole in 30 mM Tris–HCl, pH 8.0, 1 mM CaCl_2_. Except for GH43_29_-1231, the purification was achieved on Q-Sepharose fast flow (GE Healthcare) equilibrated in 30 mM Tris–HCl, pH 8.0, 1 mM CaCl_2_. The protein GH43_29_-1231 was mainly produced under insoluble form, despite the presence of the GST-Tag, and a low quantity of protein was purified on nickel-affinity column. For this particular recombinant protein, only one chromatography was done. The purified proteins were dialyzed and concentrated by ultrafiltration against 20 mM Tris‐maleate, pH 6.0, 1 mM CaCl_2_, and stored at − 80 °C. The concentration of the proteins was estimated by measurement of the absorbance at 280 nm and the use of the molar extinction coefficient calculated by ProtParam tool (https://web.expasy.org/protparam: GH43_16_-1229 101,565 M^−1^ cm^−1^, GH43_29_-1231 154,310 M^−1^ cm^−1^, GH43_10_-1233 144,550 M^−1^ cm^−1^, GH62-1234 127,130 M^−1^ cm^−1^, GH43_29_-1235 114,305 M^−1^ cm^−1^, GH62-CE6-1240 172,300 M^−1^ cm^−1^). Sodium dodecyl sulfate–polyacrylamide gel electrophoresis (SDS-PAGE) was performed using precast gels 4–15% acrylamine (Bio-Rad). Gels were stained with coomassie blue.

### Enzyme activity

WAXY-I, WAXY-RS, arabinan (from sugar beet), and linear arabinan were purchased from Megazyme and OSX was purchased from Sigma-Aldrich. WAXY-I and WAXY-RS have almost the same ratio Ara*f/*Xyl*p* (36/51 and 38/62, respectively). Insoluble substrates WAXY-I and OSX were washed with milliQ water onto the Stericup^®^ vacuum filtration system (0.22 µm pore size, polyethersulfone membrane, Sigma-Aldrich). Activities were determined at 37 °C, under mild shaking (70 rpm) by mixing 4 mL of substrate at 17.5 g/L in 20 mM Tris‐maleate, pH 6.0, 1 mM CaCl_2_, 0.01% (w/v) azide with 40 μl of an appropriate concentration of enzyme (between 10 nM to 1 µM). At specific intervals, aliquots of 500 μL were cooled on ice. For the insoluble substrates, aliquots were centrifuged at 4 °C for 5 min at 15,000*g*. 0.2 mL of soluble products released were mixed with 50 μL of 0.5 M sodium hydroxide and analyzed by high‐pressure anion exchange chromatography coupled with pulsed amperometric detector (HPAEC‐PAD) (Dionex CarboPac PA1 column) as previously described [13]. Arabinoxylooligosaccharides (AXOS) A^3^X, A^2^XX, XA^3^XX, A^2+3^XX, and XA^2+3^XX were purchased from Megazyme. Activities were determined at 37 °C, under 750 rpm shaking (Eppendorf Thermomixer), by mixing 90 μL of substrate at 1.11 mM in 20 mM Tris-maleate, pH 6.0, 1 mM CaCl_2_ with 10 μL of enzyme (ranging from 10 nM to 1 µM). Samples (0.2 mL) were collected at specific time intervals, cooled on ice, and mixed with 50 μL of 0.5 M sodium hydroxide before to be analyzed by HPAEC-PAD.

Kinetic parameters were determined on washed WAXY-I by incubating at 37 °C, under 750 rpm shaking (Eppendorf Thermomixer), 1.5 mL of substrate at various concentrations (17.5 g/L–10 g/L–5 g/L–3.33 g/L–2 g/L–1.33 g/L–1 g/L) in 20 mM Tris‐maleate, pH 6.0, 1 mM CaCl_2_, 0.01% (w/v) sodium azide with GH43_10_-1233 at 100 nM (final concentration) or GH62-1234 and GH62-CE6-1240 at 1 µM (final concentration). At specific intervals, 500 μL aliquots were cooled on ice and centrifuged at 4 °C for 5 min at 15,000*g*. Samples (0.2 mL) were mixed with 50 μL of 0.5 M sodium hydroxide and analyzed by HPAEC-PAD. Injection of samples containing arabinose, xylose, and AXOS at known concentrations was used to identify and quantify the released sugars. Specific activities are given in IU/μmol (1 IU/µmol = 1 µmol of arabinose released per minute by 1 µmol of enzyme under the experimental conditions used). These are calculated by determination of the quantity of arabinose released after 10 min of reaction by HPAEC-PAD. *k*_cat_ in s^−1^ and *K*_m_ in g/L. The *K*_m_ and *V*_max_ values were obtained by Lineweaver–Burk plots and the *k*_cat_ calculated from the *V*_max_ values. The program OriginPro was also used to fit the Michaelis–Menten representation.

The acetyl-xylan esterase activity was measured using washed WAXY-I at the concentration of 17.5 g/L. GH62-CE6-1240 at the final concentration of 1 µM was incubated with 4 mL of substrate, at 37 °C under mild shaking (70 rpm). At specific intervals, aliquots of 500 µL were cooled on ice and centrifuged at 4 °C for 5 min at 15,000*g*. 0.2 mL of soluble products released were mixed with 50 μL of 25 mM H_2_SO_4_ and analyzed using an IOTA 2 Differential Refractometer and an HPX-87H HPLC column (Bio-Rad) preceded by the corresponding guard column (Micro-Guard HPLC column protection system). The acid acetic released was eluted with H_2_SO_4_ 5 mM at 55 °C, and a constant flow rate of 0.6 mL/min. Acetic acid at known concentrations (ranging from 0.5 to 10 mM) was used as the standard. The specific activity (IU/μmol) is the amount (in µmol) of released acetic acid per minute and per µmol of enzyme.

Synergistic action between GH43_10_-1233 and GH62-1234 was measured as follows: 4 mL of washed WAXY-I at the concentration of 17.5 g/L was incubated with 40 µL of GH43_10_-1233 (final concentration 0.1 µM) and 40 µL of GH62-1234 (final concentration 1 µM) 24 h at 37 °C under mild shaking (70 rpm). Aliquots of 500 µL were collected, cooled on ice, and centrifuged at 4 °C for 10 min at 10,000*g*. 0.2 mL of soluble products were mixed with 50 μL of 0.5 M sodium hydroxide and analyzed by HPAEC-PAD.

### Three-dimensional structure modeling

We used various servers for the construction of 3D models. Each α-l-ABF characterized was modeled using each program (SWISS-MODEL, Phyre2, and RaptorX). The identification of structural template(s) comes from the initial protein primary structures from the KEGG SSDB Database [cce:Ccel_1229, cce:Ccel_1233, cce:Ccel_1234, and cce:Ccel_1240]. The SWISS-MODEL server was used to build protein models, using a template structure and target-template sequences alignments. For each selected template, a 3D protein model was automatically generated and the assessment of the model’s quality: the GMQE score and QMEAN score were calculated. GMQE (Global Model Quality Estimation) score and QMEAN score provided a quality estimation of the structural features observed for the model. GMQE score is expressed as a number between 0 and 1. QMEAN scores around zero indicate good agreement between the model structure and experimental structures.

The Phyre2 (Protein Homology/AnalogY Recognition Engine) server was also used to generate 3D models after searching for homologous sequences in the HHblits database (HMM–HMM-based lightning-fast iterative sequence search). For each model constructed, the ProQ2 quality assessment was checked [20, 21].

RaptorX was generally used to generate models for protein sequences without close homology (less than 30% sequence identity in PDB). It assigns confidence scores to evaluate the quality of the predicted 3D model: *P* value for the relative global quality (the smaller the *P* value, the higher quality the model), GDT (global distance test), and uGDT (un-normalized GDT) for the absolute global quality. For a protein with > 100 residues, uGDT > 50 and GDT > 50 are good indicators.

PyMOL (http://www.pymol.org) was used to visualize, analyze the 3D model, and to construct the figures shown in the present article.

## Results

### Modular organization and putative function of Xyl-Doc enzymes

The 14 putative cellulosomal proteins (called Xyl-Doc enzymes) encoded by the *xyl-doc* gene cluster (Ccel_1229 to Ccel_1242) all display a modular organization (Table 1) and are predicted to be involved in hemicellulose degradation. The *xyl*-*doc* gene cluster was specifically expressed when arabinoxylan, arabinose, or xylose was used as substrates, and Xyl-Doc enzymes were consequently detected by proteomic analysis in the cellulosomes produced by *R. cellulolyticum* grown in these specific culture conditions [18, 19]. Some of them, which have a catalytic module belonging to families GH43 and GH62, are annotated as α-l-arabinofuranosidases (Table 1). In the CAZy database, GH43 family contain β-xylosidases, α-l-arabinofuranosidases, arabinanases, xylanases, galactan 1,3-β-galactosidases, exo-α-1,5-l-arabinofuranosidases, exo-α-1,5-l-arabinanases, and β-1,3-xylosidases. They are organized in 37 subfamilies [23]. The *xyl*-*doc* gene cluster encodes four GH43 containing proteins: GH43_16_-1229 belongs to subfamily 16, GH43_29_-1231 and GH43_29_-1235 belong to subfamily 29, and GH43_10_-1233 belongs to subfamily 10. Their predictive functions according to the subfamily membership are reported in the Table 1. Most characterized enzymes, belonging to the subfamilies 16, 29 and 10, subfamilies found in the Xyl-Doc GH43, show some polyspecificity: β-xylosidase, xylanase, and α-l-ABF activities [23]. Only a thorough enzymatic characterization using natural substrates can allow discrimination between these activities. The catalytic modules of the two proteins belonging to the subfamily 29 (Xyl-Doc enzymes: GH43_29_-1231 and GH43_29_-1235) share 67% of identity, whereas these modules display approximatively 30% of identity with those found in other Xyl-Doc GH43 enzymes.

**Table 1:** Modular organization of Xyl-Doc enzymes and putative functions

Locus^a^	Xyl-Doc enzyme^b^	Modular structure^c^	NCBI-protein ID	Putative function^d^
Ccel_1229	GH43_16_-1229	S-GH43_16_-CBM6-DOC	ACL75585	Xylanase/α-l-arabinofuranosidase
Ccel_1230	GH10-1230	S-GH10-UNK-CBM6-DOC	ACL75586	Xylanase
Ccel_1231	GH43_29_-1231	S-GH43_29_-CBM6-DOC	ACL75587	Xylanase/xylosidase/α-l-arabinofuranosidase
Ccel_1232	CE1-1232	S-CE1-CBM6-DOC	ACL75588	Feruloyl esterase
Ccel_1233	GH43_10_-1233	S-GH43_10_-X19-CBM6-DOC	ACL75589	Xylosidase/α-l-arabinofuranosidase
Ccel_1234	GH62-1234	S-GH62-CBM6-DOC	ACL75590	α-l-Arabinofuranosidase
Ccel_1235	GH43_29_-1235	S-GH43_29_-CBM6-DOC	ACL75591	Xylanase/α-l-arabinofuranosidase
Ccel_1236	UNK-1236	S-COG3533-CBM22-UNK- DOC	ACL75592	Unknown
Ccel_1237	GH27-1237	S-GH27-UNK-CBM6-DOC	ACL75593	α-Galactosidase
Ccel_1238	GH59-1238	S-GH59-UNK-CBM6-DOC	ACL75594	α-Galactosidase
Ccel_1239	GH2-1239	S-UNK-GH2-UNK-CBM6-DOC	ACL75595	β-Galactosidase
Ccel_1240	GH62-CE6-1240	S-GH62-CBM6-DOC-CE6	ACL75596	α-l-Arabinofuranosidase/acetyl-xylan esterase
Ccel_1241	GH95-1241	S-UNK-GH95-CBM32-CBM6-DOC	ACL75597	Fucosidase
Ccel_1242	GH30-1242	S-GH30-UNK-CBM6-DOC	ACL75598	Glucuronoxylanase

For GH, CE, and CBM, the family is given by an associated number according to the CAZy database [22]. For GH43 subfamily is mentioned by a subscript number [23]. UNK: module of unknown function. X19: additional module of unknown function found associated with GH43 catalytic module in some proteins. COG3533: uncharacterized conserved module. DOC: dockerin module

*S* signal sequence, *GH* glycoside hydrolase, *CE* carbohydrate esterase, *CBM* carbohydrate-binding module

^a^*xyl*-*doc* gene cluster (loci Ccel_1229 to Ccel_1242)

^b^Notation of each Xyl-Doc enzymes according to its DNA locus and GH family

^c^Modular organization

^d^Putative function [19]

An X19 module is found associated with the GH43 subfamily 10 module of the protein GH43_10_-1233. The function of the X19 modules, which have a β-propeller fold and are found in subfamilies 9 to 14 and 36, is still unclear. They might be involved in the stabilization on the three-dimensional structure of the associated catalytic modules [23]. The whole GH43_10_-1233 protein shows 89% and 91% of identity, respectively, with uncharacterized proteins from *Ruminiclostridium papyrosolvens* DSM 2782 (EPR10800) and *Clostridium* sp BNL1100 (AEY65170), two other *Clostridium* displaying similar *xyl-doc* gene cluster organization and surrounding genes.

GH62 family only contains enzymes described as α-l-arabinofuranosidases. Two Xyl-Doc GH62-enzymes (GH62-1234 and GH62-CE6-1240) could be involved in the removal of Ara*f* decoration in arabinoxylan. The product of the gene at locus Ccel_1240 encodes an enzyme with two distinct catalytic modules (GH62 and CE6). This enzyme is predicted to have an α-arabinofuranosidase activity and an acetyl-xylan esterase activity. The GH62 catalytic module of the two GH62-Xyl-Doc enzymes are strictly identical, except for one amino-acid at position 326 (T in GH62-1234 replaced by E in GH62-CE6-1240). It is interesting to note that the adjacent CBM6 of these two catalytic modules are also strictly identical. In terms of nucleotidic sequences, the 5′-parts of these two genes show more than 97% of identity. A bifunctional cellulosomal enzyme, RjAbf62A-Axe6A, from *Ruminiclostridium josui* JCM 1788, with exactly the same modular organization and a high sequence identity (91%) was recently characterized and its role in the arabinoxylan degradation was demonstrated [24]. This enzyme is the first GH62 characterized that has an endoxylanase activity in addition to its α-l-ABF function.

**Fig. 1:**
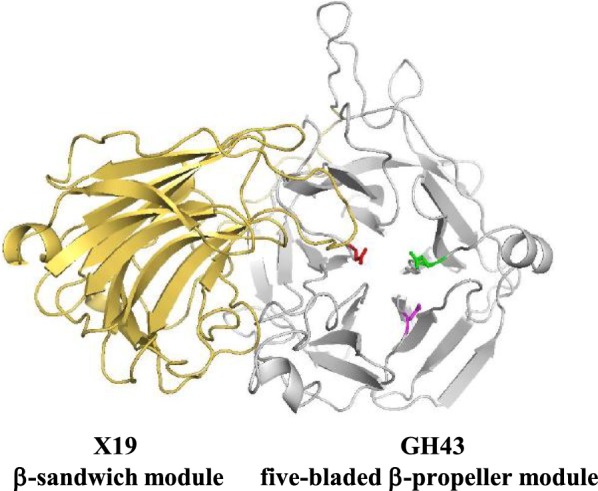
Overall structure of the N-terminal part of the mature form of GH43_10_-1233. In gray, the ribbon representation of the GH43 module with the three catalytic amino-acids: in red, the catalytic aspartate D46; in purple, the aspartate “helper” D156; and in green, the catalytic glutamate E207. The X19 module is colored in yellow

GH43 and GH62 enzymes display a five-bladed β-propeller fold (clan F). The active site is located in a deep cavity in the center of the β-propeller. They are inverting enzymes. Two conserved carboxylate residues, an aspartate and a glutamate, that act as catalytic acid and base, respectively, are required to catalyze the hydrolysis of the glycosidic bond. An additional conserved aspartate is necessary for the catalysis, though its role is not fully established. It could help to maintain the correct alignment of the general acid residue with the substrate [25]. The three conserved acidic amino-acids were found in the primary structure of the six Xyl-Doc GH43 and GH62 modules, as determined by sequence alignment. In the structural models generated with SWISS-MODEL, Phyre2, and RaptorX, these residues occupy the center of the cavity as expected. The additional X19 module of the protein GH43_10_-1233 (encompassing amino-acids 344 to 533) displays a canonical β-sandwich fold (Fig. 1). The structure of a GH43 β-xylosidase from *Geobacillus stearothermophilus* showed that the cleft of the active site is partly blocked by a loop originating from the X19 module [26]. This loop closes the cleft on one side to form the catalytic pocket. The crystal structure of HiAXHd3 (GH43 subfamily 36) from *Humicola insolens* also showed an overlap between the GH43 module and the X19 module, and the interface between these two modules bears the substrate-binding site and contributes to the overall configuration of the substrate-binding pocket [27]. It has been demonstrated that the stable folding of each module is dependent upon the other showing conformational and catalytic importance of the overlap between these two modules [27]. A loop coming from the X19 module is also found at the proximity of the catalytic cavity of GH43_10_-1233 (Fig. 1).

### Putative Xyl-Doc α-l-ABFs production and purification

To investigate the function of these six putative α-l-ABFs, the mature form of each protein (encompassing the catalytic module, the CBM6, and the dockerin module) was produced in *Escherichia coli*. Recombinant proteins were tagged with a hexa-histidine sequence at their C-terminus. Proteins were purified using an immobilized metal affinity chromatography (IMAC) followed by an anion exchange chromatography step to achieve a significantly high yield and high purity of proteins. Production of recombinant GH43_29_-1231 in *E. coli* resulted in aggregation into inclusion bodies regardless of the growth temperature and inducer (IPTG) concentration. To obtain a soluble form of the protein, a fusion with glutathione S-transferase was done. Recombinant proteins containing a GST-tag at their N-terminal extremity were often reported to have greater solubility [28]. However, the recombinant GH43_29_-1231 was still predominantly insoluble and only a very low amount of purified protein could be obtained. The purity of the proteins was checked by SDS-PAGE (Additional file 2: Figure S1) and their migration was in agreement with the molecular masses predicted from the polypeptide sequences of the recombinant forms (GH43_16_-1229: 56,281 Da; GH43_29_-1231: 81,774 Da; GH43_10_-1233: 79,932 Da; GH62-1234: 57,165 Da; GH43_29_-1235: 54,169 Da and GH62-CE6-1240, 86,290 Da).

### Putative Xyl-Doc α-l-ABFs activities on polysaccharides

To determine the substrate specificities and the functions of these six enzymes, each was tested for its capacity to degrade arabinoxylan and the released products were analyzed by HPAEC-PAD (Table 2). Wheat-flour arabinoxylan for reducing-sugar assays (WAXY-RS), a highly decorated soluble substrate (ratio Ara*f/*Xyl*p *= 38/62), was first used to analyze the nature of the released sugars and discriminate α-l-ABFs from other enzymatic functions (Table 2). The recombinant protein GH43_29_-1231 showed a β-xylosidase activity, since only xylose is released from arabinoxylan, with a relatively low specific activity at around 0.9 IU/µmol. This enzyme is the first member of this subfamily described as a β-xylosidase.

**Table 2:** Specific activities of the putative Xyl-Doc α-L-ABFs on WAXY-RS

	Specific activity^a^	Released soluble sugars^b^
GH43_16_-1229	0.191 ± 0.005	Arabinose
GH43_29_-1231	0.874 ± 0.005	Xylose
GH43_10_-1233	453 ± 13	Arabinose
GH62-1234	4.29 ± 0.14	Arabinose
GH43_29_-1235	0.767 ± 0.007	Xylose (80%)/arabinose (20%)
GH62-CE6-1240	3 ± 0.1	Arabinose

WAXY-RS wheat-flour arabinoxylan for reducing sugar assays

^a^Specific activity in IU/µmol. The data show the mean and standard deviation from six experiments

^b^Soluble sugars released, analyzed by HPAEC-PAD

The Xyl-Doc enzyme GH43_29_-1235 appears polyspecific, having both β-xylosidase and α-l-ABF activities with mainly a β-xylosidase activity on arabinoxylan. GH43 polyspecific enzymes have previously been characterized [29]. Recently, the arabinoxylan degradation profile of a GH43_29_ subfamily enzyme, AxB8 from *Clostridium thermocellum*, was shown to release xylose as the main hydrolysis product [29]. Only small amounts of arabinose are released by AxB8 such as GH43_29_-1235.

The subfamily 29 gathers various activities especially arabinan-specific arabinofuranosidases [29–31]. Nevertheless, neither GH43_29_-1231 nor GH43_29_-1235 was active on sugar-beet arabinan.

Four enzymes, GH43_16_-1229, GH43_10_-1233, GH62-1234, and GH62-CE6-1240, released only arabinose from WAXY-RS and can, thus, be considered as α-l-ABFs (Table 2). The two GH62 α-l-ABFs have the same level of specific activities from 3 to 4 IU/µmol, GH43_10_-1233 exhibits a high enzymatic activity at around 450 IU/µmol and GH43_16_-1229 is the least efficient (specific activity around 0.2 IU/µmol). Further characterization of the activities of these four α-l-ABFs was performed.

### Αctivities of the four α-l-ABFs on various polysaccharides and kinetic parameters

To further characterize the four Xyl-Doc α-l-ABFs, various substrates with different compositions were used (Table 3). Insoluble wheat-flour arabinoxylan insoluble (WAXY-I) and wheat-flour arabinoxylan for reducing-sugar assays (WAXY-RS) have almost the same Ara*f/*Xyl*p* ratios, 36/51 and 38/62, respectively. The procedure of extraction and purification of WAXY-I allow maintenance of ferulic acid cross-links and acetylation of xylose residues. WAXY-RS contains no ferulic acid cross-links or acetylated xylose residues. A poorly decorated xylan from oat spelt (OSX) with a ratio Ara*f/*Xyl*p* of 10/70 was also tested.

**Table 3:** Specific activities of the Xyl-Doc α-l-ABFs on various substrates and kinetic parameters on WAXY-I

	WAXY-RS^a^	WAXY-I	OSX	Sugar-beet arabinan
GH43_16_-1229	0.191 ± 0.005	0.028 ± 0.007^b^ *k*_cat_ and *K*_m_: ND	0.085 ± 0.056^b^	NA
GH43_10_-1233	453.7 ± 13	386 ± 37 *k*_cat_ 16.6 ± 0.6^c^ *K*_m_ 24.8 ± 0.1^c^	71 ± 1^b^	166 ± 2.8^b^
GH62-1234	4.29 ± 0.14	3.38 ± 0.56^b^ *k*_cat_ = 0.124 ± 0.002^c^ *K*_m_ = 3.735 ± 0.072^c^	4.7 ± 0.1^b^	1.3 ± 0.06^b^
GH62-CE6-1240	3 ± 0.13	2.2 ± 0.14^b^ *k*_cat_ = 0.114 ± 0.001^c^ *K*_m_ = 4.066 ± 0.068^c^	3.4 ± 0.1^b^	0.78 ± 0.12^b^

WAXY-I insoluble wheat-flour arabinoxylan insoluble, OSX oat spelt xylan. ND means not determined due to insufficient activity or lack of detectable activity

*NA* not active

^a^Specific activities from Table 2

^b^Specific activity in IU/µmol. The data show the mean and standard deviation of 6 independent experiments

^c^*k*_cat_ is given in s^−1^, *K*_m_ in g/L

Whatever the arabinoxylan used, the two GH62-α-l-ABFs display the same specific activities (Table 3). GH43_10_-1233 has the same specific activity against WAXY-RS and WAXY-I, but is around fivefold less active on OSX. GH43_16_-1229 is around ten times less active on insoluble substrates OSX and WAXY-I compared with WAXY-RS. Kinetic parameters on WAXY-I were determined, except for GH43_16_-1229, which is not active enough (Table 3). The turnover number, *k*_cat_, and the *K*_m_, found for the two cellulosomal GH62-α-l-ABFs, are in the same range as those reported for other characterized GH62-α-l-ABFs [8]. The *k*_cat_ of GH43_10_-1233 is around 150 times higher than the *k*_cat_ of the two cellulosomal GH62-α-l-ABFs and GH43_10_-1233 has a high catalytic efficiency (*k*_cat_/*K*_m_) despite an elevated *K*_m_ value.

Some α-l-ABFs are able to remove the Ara*f* decorations from arabinan. When sugar-beet arabinan was used as substrate, the GH43_10_-1233 and the two GH62-α-l-ABFs released arabinose, showing that they could recognize and hydrolyse Ara*f* decoration, whereas GH43_16_-1229 was found to be completely inactive (Table 3). Linear arabinan was degraded by none of these enzymes, indicating that none of them have an α-l-arabinanase activity or an exo-α-(1 → 5)-degradative function.

The bifunctional enzyme GH62-CE6-1240 can release acetate from WAXI-I (specific activity: 2.83 ± 0.21 IU/µmol), indicating that this enzyme is able to remove two distinct decorations of the main chain: Ara*f* and acetylation.

### α-l-ABFs activities on arabinoxylooligosaccharides

The results given above showed that the *xyl*-*doc* gene cluster encodes 4 α-l-ABFs: GH43_16_-1229, GH43_10_-1233, GH62-1234, and GH62-CE6-1240. Activities were also explored using arabinoxylooligosaccharides (AXOS) containing single Ara*f* decorations or double Ara*f* decorations (Table 4). According to the one-letter code system already published [32], we used the following nomenclature: the letter X corresponds to an unsubstituted xylose residue and A^x^ with a superscript number (x) describes a xylose residue decorated by an Ara*f* residue. If Ara*f* is α-1,2-linked, the decorated Xyl*p* residue is noted A^2^. If Ara*f* is α-1,3-linked, the decorated Xyl*p* residue is noted A^3^. If the xylodextrin contains a double decoration, the substituted xylose residue substituted is noted: A^2+3^.

**Table 4:** Activities of the Xyl-Doc α-l-ABFs on AXOS

	A^3^X^a^	A^2^XX^a^	XA^3^XX^a^	A^2+3^XX^a^	XA^2+3^XX^a^
GH43_16_-1229	NA	NA	NA	NA	NA
GH43_10_-1233	NA	NA	NA	A^3^XX/A^2^XX^b^	XA^2^XX/XA^3^XX^b^
GH62-1234	Ara*f*/X2^b^ 0.0510 ± 0.0028^c^	Ara*f*/X3^b^ 0.590 ± 0.043^c^	Ara*f*/X4^b^ 0.116 ± 0.005^c^	NA	NA
GH62-CE6-1240	Ara*f*/X2^b^ 0.0300 ± 0.0005^c^	Ara*f*/X3^b^ 0.360 ± 0.028^c^	Ara*f*/X4^b^ 0.088 ± 0.0007^c^	NA	NA

*NA* not active

^a^Substrates listed according to the nomenclature established by Fauré et al. [32] are written as follow: X2: xylobiose, X3:xylotriose, X4: xylotetraose

A^3^X: α-l-Ara*f*-(1 → 3)-β-d-Xyl*p*-(1 → 4)-d-Xyl*p*

A^2^XX: α-l-Ara*f*-(1 → 2)-β-d-Xyl*p*-(1 → 4)-β-d-Xyl*p*-(1 → 4)-d-Xyl*p*

XA^3^XX: β-d-Xyl*p*-(1 → 4)-[α-l-Ara*f*-(1 → 3)]-β-d-Xyl*p*-(1 → 4)-β-d-Xyl*p*-(1 → 4)-d-Xyl*p*

A^2+3^XX: [α-l-Ara*f*-(1 → 2)][α-l-Ara*f*-(1 → 3)]-β-d-Xyl*p*-(1 → 4)-β-d-Xyl*p*-(1 → 4)-d-Xyl*p*

XA^2+3^XX: β-d-Xyl*p*-(1 → 4)-[α-l-Ara*f*-(1 → 2)][α-l-Ara*f*-(1 → 3)]-β-d-Xyl*p*-(1 → 4)-β-d-Xyl*p*-(1 → 4)-d-Xyl*p*

^b^Products of hydrolysis analyzed by HPAEC-PAD. The data show the mean and standard deviation of six independent experiments

^c^Specific activity in IU/µmol

GH43_16_-1229 is totally inactive on the tested AXOS (Table 4), despite the high enzyme concentration used (5 µM). The Ara*f* decorations located on the non-reducing Xyl*p* residue or penultimate Xyl*p* residue from the non-reducing end in these AXOS, are not cleaved by GH43_16_-1229. We can hypothesize that this enzyme is an α-l-ABF specific of the decorations located at the reducing end of the main chain. However, in the absence of appropriate substrates commercially available, it is not possible to confirm this hypothesis. Consequently, we are also unable to provide further information with respect to its specificity for mono- or double Ara*f* decorations. Anyway, the singularity of the sugar-motif recognized by this enzyme could explain its very low specific activity on arabinoxylan.

**Fig. 2:**
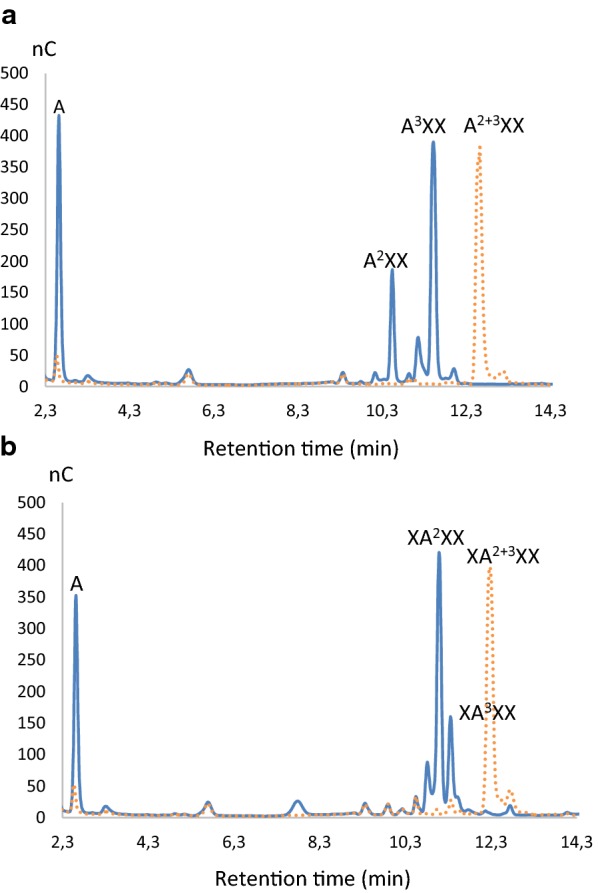
HPAEC-PAD analysis of A^2+3^XX (**a**) and XA^2+3^XX (**b**) hydrolyzed by GH43_10_-1233 after 24 h. The main products of the reaction were identified according to the retention times of standards: Arabinose (A), A^2^XX and XA^3^XX. **a** Standards used allowed us to identify A and A^2^XX, the structure of the third main product of the reaction, A^3^XX; was deduced from the chromatogram. **b** Standards used allowed us to identify A and XA^3^XX, the structure of the third main product of the reaction XA^2^XX was deduced from the chromatogram. In red dotted lines, the substrate alone, entirely degraded after 24 h

GH43_10_-1233 cleaves Ara*f* decorations from internal di-substituted xylodextrins (Table 4) and is inactive on mono-substituted substrates. This enzyme can cleave α-(1 → 2) and α-(1 → 3). HPAEC-PAD analysis of the products, from A^2+3^XX and XA^2+3^XX hydrolysis showed that the main products, apart from Ara*f*, are, respectively, A^3^XX and XA^2^XX indicating that, depending upon the position of the double substitution in the AXOS, the cleaved linkage varies (Fig. 2a, b).

The two GH62-α-l-ABFs encoded by the *xyl*-*doc* gene cluster were found to be active on polysaccharides and mono-substituted AXOS. For both enzymes, a lower specific activity was found on arabinoxylobiose A^3^X and on arabinoxylotetraose XA^3^XX, maybe because the preferential cleaved glycosidic bond is α-(1 → 2). However, these enzymes seem to prefer polysaccharides. Indeed, the specific activity is almost 100-fold higher on WAXY-I, WAXY-RS, and OSX than on AXOS. The same specific activity was observed on WAXY and OSX despite a lower amount of Ara*f* decorations in OSX. This observation can probably be explained by the fact that, in OSX, the xylose backbone predominantly harbors Ara*f* mono-substitutions. This hypothesis might also explain the lower specific activity of GH43_10_-1233 on OSX, which specifically recognizes double Ara*f* decorations.

### Synergistic action between GH43_10_-1233 and GH62-1234

**Fig. 3:**
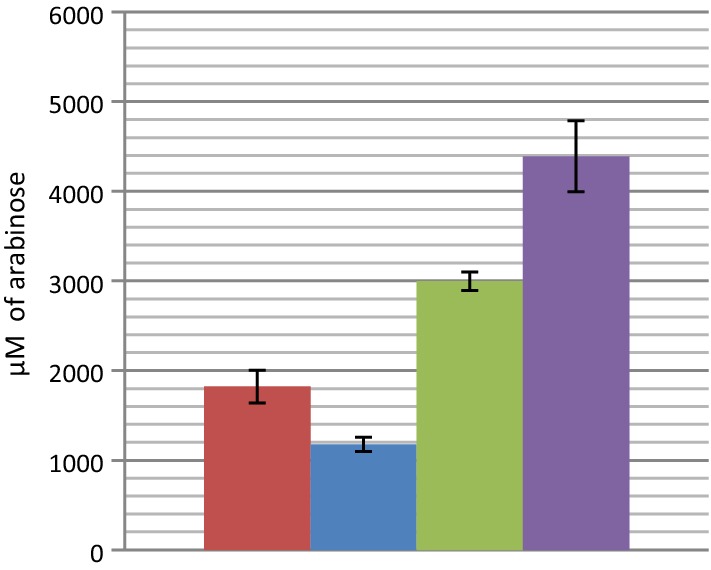
Synergistic action between GH43_10_-1233 and GH62-1234. WAXY-I was hydrolyzed at 37 °C during 24 h. Quantities of arabinose released by GH43_10_-1233 alone (in red), by GH62-1234 alone (in blue) or by a mixture of the two (in purple), were determined by HPAEC-PAD chromatography. The theoretical quantity of arabinose produced by both enzymes is shown in green. The data show the mean and standard deviation of four independent experiments

According to their modes of action, GH43_10_-1233 and GH62-1234 should act in synergy. Indeed, the single Ara*f* substitutions (O2- or O3-α-linked) generated by GH43_10_-1233 on polysaccharides could be recognized and cleaved by GH62-1234. This hypothesis was confirmed, as shown in Fig. 3. A synergy factor of 1.53 (ratio between the experimental quantity of arabinose released by the mixture of GH43_10_-1233 and GH62-1240 and the calculated sum of arabinose released by each enzyme alone) was found between these two enzymes on WAXY-I.

### Three-dimensional structure modeling

**Fig. 4:**
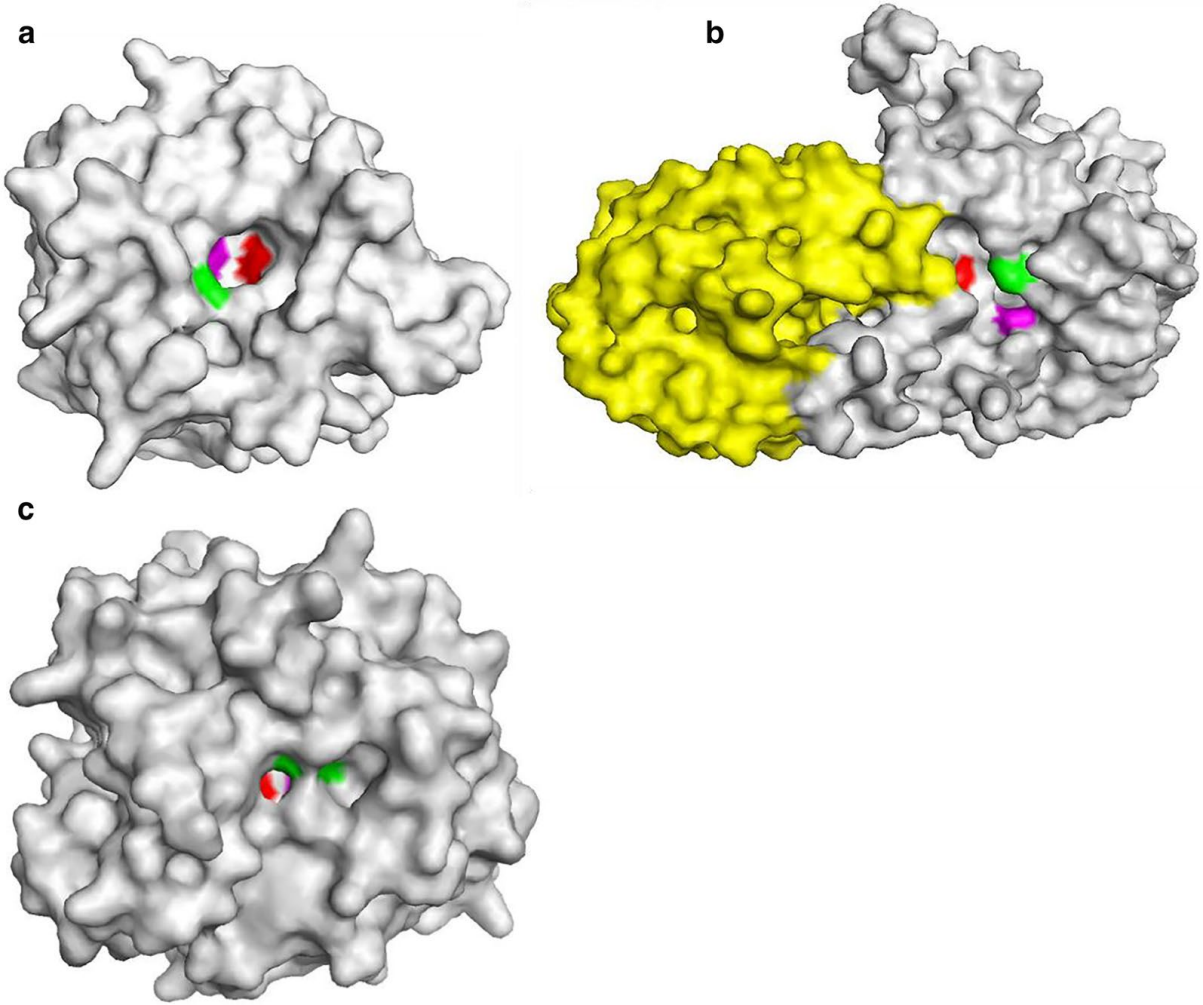
Overall structure modeling of GH43_16_-1229, GH43_10_-1233 and GH62-1234. Models established using the server Phyre2 are shown in this figure. The ProQ2 (Phyre 2 server) quality assessment was checked for each model. Quality scores obtained with SWISS-MODEL and RaptorX were determined for each model. **a** The 3D structure of the catalytic module of the protein GH43_16_-1229 was built using the 3D structure of Ct43Araf (PDB ID: 5a8d.1.A) from *Clostridium thermocellum.* In red, the catalytic aspartate D40; in purple, the aspartate “helper” D159,; and in green, the catalytic glutamate E208. (SWISS-MODEL quality scores: GMQE = 0.8, QMEAN = − 2.33; RaptorX quality scores: *P* = 5.6710^−15^, uGDT(GDT) = 273 (88). **b** The structure of the N-terminal part (GH43-X19) of GH43_10_-1233 was modeled on the basis of the 3D structure of HiAXHd3 (PDB ID: 3zxl.A) from *Humicola insolens.* In red, the catalytic aspartate D46; in purple, the aspartate “helper” D156; and in green, the catalytic glutamate E207. The X19 module is colored in yellow. (SWISS-MODEL quality scores: GMQE = 0.7, QMEAN = − 4.5; RaptorX quality scores: *P* = 3.3610^−19^, uGDT(GDT) = 325 (61). **c** For both GH62 catalytic modules (GH62-1234 and GH62-CE6-1240), the template used was the GH62-α-l-ABFs from *Coprinopsis cinerea* (PDB ID: 5b6s.1.A). In red, the catalytic aspartate D57; in purple, the aspartate “helper” D163; and in green, the catalytic glutamate E213. (SWISS-MODEL quality scores: GMQE = 0.7, QMEAN = − 3.94; RaptorX quality scores: *P* = 1.1210^−10^, uGDT(GDT) = 210 (67)

Computational methods for protein structure modeling (SWISS-MODEL, Phyre2 and RaptorX) were used to shed some light on our biochemical results. The 3D structure of the catalytic module of the protein GH43_16_-1229 (residues 33–314 based on KEGG SSDB Database, cce:Ccel_1229) was modeled to refine the understanding of its mode of action. Structural models were built using the 3D structure of Ct43Araf (PDB ID: 5a8d.1.A) (subfamily 16) from *Clostridium thermocellum,* which shares 64% sequence identity with the GH43 module of GH43_16_-1229. GH43_16_-1229 displays a five-bladed β-propeller structure (each propeller formed by four antiparallel β-strands) and its molecular surface reveals a narrow and deep pocket for the accommodation of only one Ara*f* residue. The three putative conserved catalytic amino-acids were identified by multiple alignment (D40, D159, and E208) (Fig. 4a). Similarly to GH43_16_-1229, Ct43Araf has the ability to catalyze the hydrolysis of terminal arabinosyl residues in arabinoxylans [33, 34]. Ct43Araf hydrolyses the Ara*f* decorations (α-1,2 or α-1,3) located at the non-reducing extremity of arabinoxylans, whereas our biochemical results suggest, for GH43_16_-1229, an action at the reducing extremity. According to the narrowness of the catalytic pocket, GH43_16_-1229 could be specific of Ara*f* mono-substitutions. GH43_16_-1229 and Ct43Araf act only on arabinoxylans and are unable to cleave off arabinose decorations from arabinan. These biochemical results are consistent with the structure of Ct43Araf which reveals the presence of a substrate-binding cleft designed to interact with arabinoxylan but unable to accommodate the nonlinear backbone of arabinan [33, 34].

The N-terminal part of GH43_10_-1233, composed of two modules (GH43: residues 35 to 300 and X19: residues 344 to 533), was modeled using the 3D structure of HiAXHd3 (PDB ID: 3zxl.A) (subfamily 36) from *Humicola insolens* which shares 37% identity with the GH43-X19 part of GH43_10_-1233 (Figs. 1 and 4b). HiAXHd3 is, like GH43_10_-1233, highly specific for substrates containing double Ara*f* substitutions [27]. The structure of the catalytic cavity of GH43_10_-1233 shows a shallow but extended pocket able to accommodate two Ara*f* residues. The three putative catalytic amino-acids (D46, D156, and E207) are localized, according to our model, around the groove inside which Ara*f* decorations might interact (Fig. 4b). Like other GH43 proteins carrying an additional X19 module, the 3D model shows structural connections between these two modules.

The primary structures of the two GH62 catalytic modules from GH62-1234 (residues 35 to 295 based on KEGG SSDB Database, cce:Ccel_1234) and GH62-CE6-1240 (residues 35 to 294 based on KEGG SSDB Database, cce:Ccel_1240) are strictly identical except for one residue located at position 236. For both GH62 catalytic modules, the template used was the GH62-α-l-ABFs from *Coprinopsis cinerea* (PDB ID: 5b6s.1.A) [35]. The three-dimensional modeling revealed, as expected for an enzyme belonging to the clan F, a structure in five-bladed β-propeller with a narrow pocket, carrying the three putative catalytic amino-acids, identified by multiple alignment. GH62-α-l-ABFs from *C. cinerea* accommodates a single Ara*f* residue [35]. The narrowness of the catalytic cavities of both GH62-1234 and GH62-CE6-1240 also suggests an interaction with a single Ara*f* substitution, in agreement with the enzymatic characteristics described above (Fig. 4c). The serine–histidine–glycine (SHG) motif conserved within the GH62 family [8, 36, 37] and in some GH43 members was also found in the four *R. cellulolyticum* α-l-ABFs described in this paper. In the arabinase, BsArb43B from *Bacillus subtilis*, the histidine, at the bottom of the catalytic cavity, coordinates a calcium ion and plays a structural role [25].

## Discussion

Cellulosomes of *Ruminiclostridium cellulolyticum* are extracellular multi-enzyme machineries, which efficiently degrade plant cell wall polysaccharides. 62 annotated CAZymes contain a type I-dockerin module and are integrated into the cellulosomes through interactions with the scaffolding protein CipC. Many cellulosomal enzymes display activity against cellulose, hemicelluloses (arabinoxylans and xyloglucans), and pectins, and it was shown, by transcriptomic and proteomic analyses, that the composition of *R. cellulolyticum* cellulosomes is modulated according to the growth substrates [13, 19, 38, 39]. Genes encoding cellulases, especially those gathered in the *cip*-*cel* cluster, are regulated by carbon catabolite repression, while the expression of most of the genes encoding other CAZymes and accessory enzymes is activated via two-component regulation systems as it was shown for the *xyl*-*doc* gene cluster [17–19, 39]. This 32-kb gene cluster encodes modular CAZymes putatively involved in the degradation of hemicelluloses components such as arabinoxylan which can constitute up to 40% of the total plant cell wall dry mass depending on the plant species, especially in the cell walls of wheat grain [40]. *xyl*-*doc* gene expression is induced by the presence of straw, and arabinoxylan, and XydR, a response regulator acts as an activator of the transcription of this operon. The complete degradation of arabinoxylans requires endo-β-1,4-xylanases, β-xylosidases, and several accessory enzymes such as α-l-ABFs. The role of α-l-ABFs in the plant cell wall degradation is crucial, because these enzymes enhance the hydrolytic rate of endo-β-1,4-xylanases, particularly in substrates from agricultural residues such as wheat straw, corn fiber, and rice straw. α-l-ABFs are, therefore, promising tools in various processes like, for instance, the production of bioethanol [11]. *R. cellulolyticum* cellulosomal α-l-ABFs are exclusively encoded by the *xyl*-*doc* genes at loci Ccel_1229, Ccel_1233, Ccel_1234, and Ccel_1240 [19]. These enzymes are members of family GH43 and family GH62, and each carries a CBM6.

CBM6s are found in various CAZymes: arabinofuranosidases, xylanases, acetyl-xylan esterases, endoglucanases, agarases, mannanases, and glucanases [23, 41]. CBM6s interact with diverse carbohydrate targets. Some of them are able to interact with internal residues within a polysaccharide chain and have multiple binding subsites, other recognize a single sugar residue. The biochemical properties and structural analysis of the CBM6 of the Xyl-Doc enzyme GH59-1238, a putative α-galactosidase, demonstrated an interaction with a single xylose residue located at the extremity of a xylan chain or Xyl*p* decorations present on xyloglucans [41]. This module is unable to interact with glucose, mannose, arabinose, and galactose, and the same substrate specificity has been found for the CBM6 of the Xyl-Doc putative α-galactosidase GH27-1237 [41]. The CBM6 modules of these two enzymes, probably involved in the degradation of galacto(gluco)mannan, share 93% of sequence identity. The primary structure of the CBM6s of the α-l-ABFs studied in this paper diverge from the two CBM6s formerly characterized (only 73% of identity) and it has been suggested by Abbott et al., that some of these modules might exhibit different binding specificities [41]. CBM6s are often linked to GH43 catalytic modules belonging to subfamilies 15, 16, 29 and 2. The case of GH43_10_-1233 whose catalytic module belongs to subfamily 10, but exhibits a CBM6 is, thus, unusual. CBM6s are also not universal in GH62 enzymes, since only 2% of these enzymes are appended with a CBM6 [8].

The biochemical characterization of the putative Xyl-Doc α-l-ABFs showed that four are true α-l-ABFs and belong to the type-B. Indeed, type-B α-l-ABFs show activity from side chains of arabinan or arabinoxylan polysaccharides and decorated oligosaccharides [8]. According to the biochemical data obtained and the three-dimensional structure models, we proposed a specific mode of action for each of them.

GH43_16_-1229, which belongs to the subfamily 16 of the GH43, encoded by the gene at locus Ccel_1229, has a very low specific activity on arabinoxylan and oat spelt xylan, and could be involved in the degradation of some particular Ara*f* motifs located at the reducing-end extremity of the xylan backbone. We have renamed this enzyme RcAbf43A. Like Ct43Araf from *Clostridium thermocellum,* RcAbf43A has a strict selectivity for arabinoxylans and cannot degrade Ara*f* decorations in arabinans [34]. The previous proteomic and transcriptomic analyses have shown that the gene at locus Ccel_1229, the first gene of the operon, is highly expressed in corn fiber and that RcAbf43A is abundantly found in cellulosomes when natural complex plant cell wall materials are used as growth substrates [18, 19, 39]. This enzyme, despite its low activity, might play a crucial role in arabinoxylan degradation due to its particular mode of action. Its involvement may take place in the first stage of the hemicelluloses degradative process. RcAbf43A acts on Ara*f* decorations located on chain extremities, probably more easily available than the internal decorations due to the presence of ester cross-linkage and the other surrounding polysaccharides, and may release few molecules of arabinose which in turn could act as inducers of the expression of the *xyl*-*doc* genes. Thus, XydS, the histidine kinase of the two-component system (XydS/XydR), could sense this inducer and activate the regulator XydR. The *xyl*-*doc* operon is, thus, highly expressed allowing the massive production of the Xyl-Doc enzymes for a complete degradation of arabinoxylans.

GH43_10_-1233 renamed RcAbf43A_d2,3_ is an α-l-ABF able to convert the double Ara*f* substitutions present on arabinoxylan and arabinan into single O2- or O3-linked decorations. The possibility to accommodate two Ara*f* residues is consistent with the 3D model showing a shallow-binding cleft adjacent to the active site pocket. Up to now, three GH43 α-l-ABFs specific of the double Ara*f* substitutions have been characterized, one from *Bifidobacterium adolescentis* (BadAbf43A, subfamily 10), one from *Humicola insolens* (HiAXH_d3_ subfamily 36), and one from *Chrysosporium lucknowense* C1(Abn7 subfamily 36) [27, 42–44]. They exclusively cleave α‐(1 → 3)‐Ara*f* residues linked to bisubstituted Xyl*p.* In this respect RcAbf43A_d2,3_ of *R. cellulolyticum* has an unusual behavior, since the cleaved linkage can be either α‐(1 → 3) or α‐(1 → 2) depending upon the position of the double substitution along the xylan backbone. To our knowledge, RcAbf43A_d2,3_ is the first enzyme reported to have such an activity pattern. Its specific activity on arabinoxylan and on arabinan is rather high, and despite a *K*_m_ relatively high 24.8 g/L, this enzyme was found to be the most efficient *R. cellulolyticum* cellulosomal α-l-ABF, with a turnover value of 16.6 s^−1^ on arabinoxylan. Furthermore, it is active on highly decorated substrates such as WAXY-I or WAXY-RS, OSX, a less substituted substrate, arabinan, and arabinoxylooligosaccharides. This protein is massively found in cellulosomes purified from growth culture on straw [18]. Its substrate specificity and mode of action make this enzyme essential for the degradation of both arabinoxylan and arabinan, two substrates displaying a high content in bisubstituted xylosyls.

The two cellulosomal GH62-enzymes, products of genes at loci Ccel_1234 and Ccel_1240, found in the cellulosomes of *R. cellulolyticum* release single Ara*f* decorations, α-(1 → 2) or α-(1 → 3) linked, present on arabinoxylan, sugar-beet arabinan and arabinoxylooligosaccharides. Moreover, the bifunctional enzyme, the product of gene at locus Ccel_1240, contains an additional CE6 module able to cleave off acetyl substitutions present on WAXY-I, and, thus, harbors an acetyl-xylan esterase activity. Several plant cell wall polysaccharides in hardwood, softwood, or annual plants are esterified with acetic acid and carbohydrate esterases (CEs) catalyze the deacylation of these substrates and thereby facilitate the action of GHs. Presently, the CAZy database contains 16 CE families. The family CE6 is composed of only few characterized enzymes, all of them having an acetyl-xylan esterase activity. According to its mode of action, GH62-1234 was renamed RcAbf62A_m2,3_ and GH62-CE6-1240 was renamed RcAbf62B_m2,3_Axe6. The recently characterized bifunctional enzyme from *R. josui* RjAbf62A-Axe6A harbors an acetyl-xylan esterase activity with a specific activity of 0.0170 ± 0.0002 IU/µmol on WAXY-I [24]. Although the two enzymes show 91% of identity, this activity is relatively low compared to the acetyl-xylan esterase activity of the homologous *R. cellulolyticum* RcAbf62B_m2,3_Axe6 determined to be 2.83 ± 0.21 IU/µmol. Nevertheless, the GH62 module of RjAbf62A-Axe6A is around 50-fold more active on insoluble arabinoxylan and this enzyme is, in addition, polyspecific. Indeed, the GH62 module of RjAbf62A-Axe6 also has an endoxylanase activity in addition to the α-l-ABF activity typically found in all other GH62 enzymes.

The topology of the active site of the GH62 module, of both RcAbf62A_m2,3_ and RcAbf62B_m2,3_Axe6, modeled in this study is in good agreement with our biochemical data. The deep and narrow catalytic cavity in the center of the β-propeller is likely to accommodate only a single sugar. These two modular enzymes RcAbf62A_m2,3_ and RcAbf62B_m2,3_Axe6 have identical N-terminal moiety (GH62-CBM6) primary structure except for one amino-acid located far away from the catalytic cavity at the end of the GH62 module. The DNA sequence of the corresponding genes also shows a very high identity (97%) suggesting a gene duplication. To utilize the broad range of vegetal substrates, cellulolytic bacteria have to adapt and diversify their enzymatic arsenal by gene duplications and/or horizontal gene transfers, thereby generating novel catalytic properties.

A synergistic action was demonstrated when RcAbf62A_m2,3_ and RcAbf43A_d2,3_ are mixed together with arabinoxylan, leading to a 1.5-improvement. Similarly, it would be interesting to assay the synergy of the characterized α-l-ABFs with other GHs, especially endo-β-1,4-xylanases. Indeed, it was already known that substrates containing large amounts of arabinoxylan are not easily cleaved by endoxylanases without prior or simultaneous incubation with arabinofuranosidases [45, 46]. The potentially synergistic effect of these enzymes towards arabinan should also be explored.

## Conclusions

**Fig. 5:**
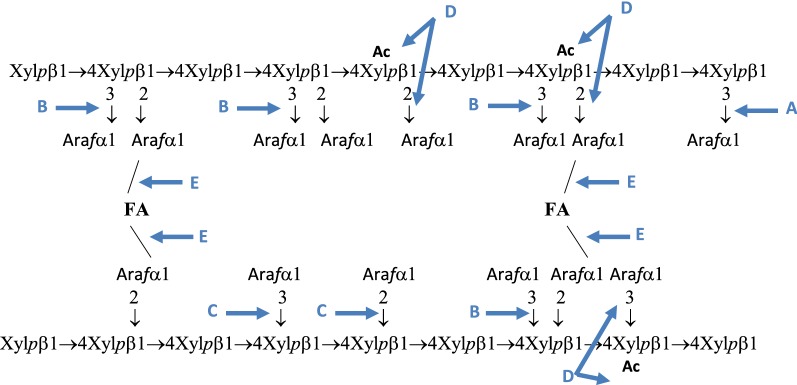
Representative arabinoxylan structure and the sites of cleavage by the accessory Xyl-Doc enzymes. Linear backbone of β-(1 → 4)-linked d-xylopyranosyl units (Xyl*p*) decorated by α-l-arabinofuranosyl (Ara*f*) α-(1 → 2) or α-(1 → 3)-linked. Acetyl substitutions (Ac) of xylose residues are also found. Some of Ara*f* residues are linked to ferulic acids (FA) allowing the formation of arabinoxylan–arabinoxylan cross-links. A: linkage putatively cleaved by RcAbf43A formerly named GH43_16_-1229. B: linkage cleaved by RcAbf43A_d2,3_ formerly named GH43_10_-1233. C: linkage cleaved by RcAbf62_m2,3_ formerly named GH62-1234. D: linkages cleaved by RcAbf62B_m2,3_Axe6 formerly named GH62-CE6-1240. E: linkage cleaved by the putative feruloyl esterase product of the gene at locus Ccel_1232

The results of the present study have allowed us to analyze the role of the α-l-ABFs encoded by the *xyl*-*doc* cluster. They are involved in hemicellulose degradation and more particularly in arabinoxylan degradation, but also in the degradation of arabinan a component of the type I rhamnogalacturonan. Complete enzymatic degradation of arabinoxylan requires the action of debranching enzymes, also called accessory enzymes, α-l-arabinofuranosidases, feruloyl esterases, acetyl-xylan esterases, and α-l-glucuronidases. Four Xyl-Doc enzymes act as α-l-ABFs, with complementary modes of actions. Together, they can remove all the Ara*f* decorations, both at chain extremities and within the xylan backbone (Fig. 5). Moreover, the bifunctional enzyme RcAbf62B_m2,3_Axe6 removes the acetyl substituent, thereby impeding the enzymatic degradation of plant cell wall polysaccharides by GHs targeting the main chain. Arabinosyl decorations in hemicelluloses and pectins participate in the cross-linking within the plant cell wall polysaccharides and, thus, α-l-ABFs can also favor the activity of feruloyl esterase by their capacity to unpack the plant cell wall polymers [47]. Indeed, synergistic action between α-l-ABFs and ferulyol esterases, another category of accessory enzymes, has previously been shown [46]. A putative feruloyl esterase is encoded by a gene present in the *xyl*-*doc* cluster (locus Ccel_1232). This enzyme may also play a pivotal role in the degradation of arabinoxylans and pectins (Fig. 5). Oncoming studies of the yet uncharacterized Xyl-Doc enzymes will certainly increase our understanding of the degradative efficiency of *R. cellulolyticum* towards plant cell wall polysaccharides.

## Additional files

**Additional file 1: Table S1.** Sequence of the primers.

**Additional file 2: Figure S1.** Purified recombinant putative α-l-ABFs. Purified proteins (about 2 µg each) were loaded on precast SDS-PAGE 4–15% of acrylamide, and stained with Coomassie Blue.

## Data Availability

All data generated or analyzed during this study are included in this published article and its additional file.

## Abbreviations

α-l-ABF: α-l-arabinofuranosidase
Ara*f*: arabinofuranose
AXH: arabinoxylan-arabinofuranohydrolase
AXOS: arabinoxylooligosaccharide
CBM: carbohydrate-binding module
CE: carbohydrate esterase
GH: glycoside hydrolase
OSX: oat spelt xylan
WAXY-I: insoluble wheat-flour arabinoxylan
WAXY-RS: wheat-flour arabinoxylan for reducing-sugar assays
Xyl*p*: xylopyranose
