# Supplementary material for: The xyl-doc gene cluster of Ruminiclostridium cellulolyticum encodes GH43- and GH62-α-l-arabinofuranosidases with complementary modes of action
Source: Biotechnol Biofuels. 2019 Jun 10;12:144. doi: 10.1186/s13068-019-1483-y (PMC6556953; doi:10.1186/s13068-019-1483-y)
Supplement: Supplementary file 1 — Additional file 1: Table S1. Sequence of the primers. [file 13068_2019_1483_MOESM1_ESM.docx]

**Additional file : Table S1 :** Sequence of the primers

| Locus (a) | Primers name | Sequence |
| --- | --- | --- |
| **Ccel_1229** | 1229pETdir  1229pETrev | TTTCATATGGACTATCCCATATTTTACCAGAGG  TTTCTCGAGTAATTCAGCAGGAAATTGGGTCC |
| **Ccel_1231** | 1231pGexdir  1231pGexrev | TATAGG ATCCTAGAC AAT CCA ATA GTG CAG ACTTT  TATACTCGAGTCAGTGGTGGTGGTGGTGGTGTAAACTAGGAAT TGT AC CAAG CAG |
| **Ccel_1233** | 1233pETdir  1233pETrev | TTTCATATGTGGCAGTCTGATAATGACAACGG  TTT CTCGAGCCCTGGAAACTCGGTAATTAGC |
| **Ccel_1234** | 1234pETdir  1234pETrev | GGAATTCCATGGCAAACCCAAATCCGTCATG  CCGACTCGAGCGCCTGAGCAGGGAATTTTG |
| **Ccel_1235** | 1235pETdir  1235pETrev | TTTCATATGGCAGATAACCCGATTGTG  TTTCTCGAGAATAATACCTAACAGAAGTTTCTT |
| **Ccel_1240** | 1240pETdir  1240pETrev | TATCCATGGCAAACCCAAATCCGTCATGG  TTTCTCGAGCCATTTCAGTGCCTGAATCATT |

(a) loci of the xyl-doc genes encoding putative α-L-Arabinofuranosidases
