# Supplementary figures and images for: The xyl-doc gene cluster of Ruminiclostridium cellulolyticum encodes GH43- and GH62-α-l-arabinofuranosidases with complementary modes of action
Source: Biotechnol Biofuels. 2019 Jun 10;12:144. doi: 10.1186/s13068-019-1483-y (PMC6556953; doi:10.1186/s13068-019-1483-y)

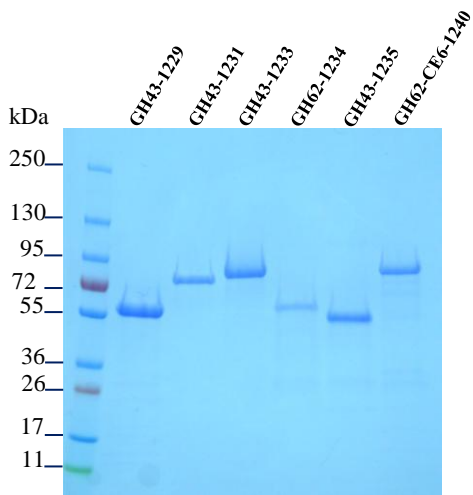

Supplement: Supplementary file 2 — Additional file 2: Figure S1. Purified recombinant putative α-l-ABFs. Purified proteins (about 2 µg each) were loaded on precast SDS-PAGE 4–15% of acrylamide, and stained with Coomassie Blue. [file 13068_2019_1483_MOESM2_ESM.pdf]
